# Mental Health Literacy of Australian Youth Sport Coaches

**DOI:** 10.3389/fspor.2022.871212

**Published:** 2022-04-12

**Authors:** Matt A. Moore, Jerry F. Reynolds, Jessica Durand, Kristin Trainor, Gina Caravaglia

**Affiliations:** ^1^Department of Social Work, Ball State University, Muncie, IN, United States; ^2^Alliance of Social Workers in Sports, Westfield, IN, United States; ^3^Department of Psychological Sciences, Ball State University, Muncie, IN, United States

**Keywords:** youth sport, Australia, mental health literacy, mental health, athletic coaching

## Introduction

Sport is an integral part of Australian culture and comprises a significant portion of time for Australian youth. Within Australia, 73.4% of children reported participating in sport once a week in the latest AusPlay survey (Australian Sports Commission, 2016). Sport historically yields positive health benefits for children and adults. The positive benefits of sport can permeate Australian society. For Australian youth, sport improves mood, enhances neurological functioning, combats chronic disease, and improves biopsychosocial development (Australian Sports Commission, 2016). However, recent national inquiries challenged this exclusively positive conception of sport in Australia, finding sports are institutions that can conceal instances of mental health challenges (Australian Human Rights Commission, 2020). Researchers found ranges of psychiatric disorders in youth athletes ranges from 4 to 68% (Elbe and Nylandsted Jensen, 2016).

In response to institutional enquiries into mental health within sport, Australian sporting bodies moved to implement mental health first aid programs and initiatives such as the Australian Institute of Sport (Australian Institute of Sport, 2019) Community Custodians program and the AIS mental health referral network. This opinion piece reflects on these programs and explores ways to heighten our awareness of youth athlete mental health, with an emphasis on the role a coach and sport organizations play in identifying and supporting youth athletes in their efforts to address mental health needs.

## Understanding Mental Health Context in Sport

Adolescence marks significant hormonal, physical, and social changes, making it a critical period for transition and development (Mazzer and Rickwood, 2013). The marked changes adolescents experience during this transitional period can cause psychosocial stress, which can lead to the development of mental illness (Romeo, 2013). In addition to general risks, sport poses some unique mental health risks to the youth population. These factors can include: team dynamics, coaches, family and institutional pressure, injury, and performance pressures (Moore, 2016). Leaders must be aware of the general vulnerabilities and unique sport factors that could impact youth athletes. Without appropriate education, intervention, and access to support services, a range of problems can occur across an individual's life course (Hughes and Leavey, 2012).

Sport-based settings show to be an important vehicle for mental health literacy and education (Hurley et al., 2018). In an Australian-based study, a mental health literacy program run with parents of young athletes, reported improvements to recognizing signs and symptoms of mental health concerns. The study also acknowledged the role of mental health literacy in providing avenues to support service acquisition (Hurley et al., 2018). Similar international studies found comparable results (Bu et al., 2020; Hurley et al., 2021).

## Coaches as a Method of Intervention

Within the ecological systems framework, coaches play a significant role in creating a safe environment for youth athletes to grow and develop. Their attitudes toward mental toughness can either hinder or promote help-seeking behaviors and overall mental health (Mazzer and Rickwood, 2013; Moreland et al., 2018). Coaches identify the need to support mental health within the scope of their position, which demonstrates an increasing expectation that coaches understand strategies for supporting/developing mental health support (Mazzer and Rickwood, 2013; Murphy and Sullivan, 2021). An Australian study into coach attitudes found they rarely engaged with young people around mental health concerns (Mazzer and Rickwood, 2013). Another concern is Australian coaches' perceptions about mental health as only existing within their purview when it negatively impacts athletic performance (Sebbens et al., 2016).

Literature examining coaches and mental health literacy is still emerging. One such study of Canadian intercollegiate coaches found a negative correlation between age and mental health literacy (Sullivan et al., 2019). Another study by Kroshus et al. (2019), examining high school coaches, identified older coaches as less likely to assist athletes with their mental health concerns. When examining years of experience coaching and mental health literacy, a United Kingdom study found coaches with 1 year of experience had higher mental health literacy, than coaches with 2 or more years of coaching experience (Gorczynski et al., 2020). This finding may be attributed to newer coaches being less likely to have been influenced by the sporting environment. By contrast, a United States study of collegiate track and field and cross-country coaches found no significant correlation between years of coaching experience and mental health literacy (Hegarty et al., 2018).

Among other factors influencing mental health literacy is personal experience with mental illness. Studies concerning the general population consistently find a positive correlation between personal experience with mental illness and increased mental health literacy (Mendenhall and Frauenholtz, 2013; O'Connor and Casey, 2015). There is current focus on the role a coach's mental health literacy has on an athlete's mental health (Norris et al., 2017). Several studies measured the prevalence of mental illness among coaches, with findings ranging from 15 to 55% of coaches having experience with mental illness (Kim et al., 2020; Smith et al., 2020). The identification of coaches having personal experience with mental illness is important because significant literature supports the utility of using lived experience to connect with others within the mental health space (White et al., 2020).

## Discussion

In the context of youth sports, both athletes and coaches interact with multiple systems in which they may encounter vulnerabilities, including challenges with mental health. Coaches play an important role in connecting athletes to the resources they may need to be successful both on and off of a playing surface (Mazzer and Rickwood, 2013; Moreland et al., 2018). This extends to mental health literacy. Athletes experiencing a mental health concern are not always able to self-identify the challenge, but community supports (Jorm, 2012), like coaches and sport organizations, can be a front-line defense to help support awareness and action for the impacted individual. Coaches acknowledged identifying needs related to mental health amongst their athletes is within their scope of work (Mazzer and Rickwood, 2013; Brown et al., 2017; Liddle et al., 2019). Such acknowledgment lends to the possibility of building continued mental health literacy within the sporting environment. This does not mean coaches should provide mental health services; rather, they should be in a position to identify and make proper connections (Weber et al., 2021).

While acknowledging the role of a coach is a start, it is the opinion of these authors that coaches and youth sport organizations must do more to support youth athletes in combatting mental health needs. While Australian youth sport agencies require the majority of coaches to receive mental health training, we fear the impact of mental health literacy programs may not be sustained and beneficial unless sporting norms and environments experience sustained change. For example, there is a large difference between knowing about mental health and taking action to address is it. There is also a large difference between taking action on mental health because it impacts an athlete's competitive edge vs. their larger life picture.

While mental health first aid and community custodian programs are a strong starting point, the issue of youth athlete mental health is not as simple as no longer concealing its existence. Instead, sport organizations must promote the mental health literacy of youth athletes through a series micro and macro efforts (e.g., direct practice, community organizing, advocacy, policy development, education, and research; Moore and Gummelt, 2018). Youth sport organizations achieve these aims by working with an interprofessional network (e.g., working collaboratively with sport psychologists, sport social workers, athletic trainers, sport administrators, and other licensed mental health professionals).

At the micro level, sport organizations must emphasize and deliver interventions that increase mental health literacy and assist youth athletes in accessing quality treatment and becoming empowered advocates for their own care (Mendenhall and Frauenholtz, 2013). This begins with detailed knowledge of mental health symptoms and disorders, diagnosis and treatment, systemic issues related to sport identity and culture, and developmental factors (Gorczynski et al., 2020). Interprofessional services also capture the needs of individuals involved in sport through various lenses. These efforts allow youth sport organizations to establish pathways to care, especially clinical care with higher level of expertise theoretically, diagnostically, and through intervention.

At the macro level, youth sport organizations can support mental health literacy by providing community education and raise awareness of mental health stigma and realities (Mendenhall and Frauenholtz, 2013) within sport. This must be done through sound pedagogical approaches that emphasize mental health literacy consistently to youth athletes and members of their ecological systems (Gorczynski et al., 2020). This includes examining strategies for effectively and efficiently communicating about mental health risks “through various media campaigns, traditional methods, and innovative technology” (Mendenhall and Frauenholtz, 2013, p. 367). These approaches consider individual and cultural differences associated with particular sports, personal factors, cultural components, environmental determinants, and appropriate formats for capturing the desired target population.

“These mental health literacy interventions empower clients and demonstrate respect for self-determination” (Mendenhall and Frauenholtz, 2013, p. 367). They demonstrate a powerful cultural awareness, commitment to social diversity, and a call to social action. These increased efforts would allow researchers to more holistically explore other factors that contribute to the mental health literacy views of coaches and youth sport organizations. Additionally, given the challenges acknowledged by scholars in retention of mental health knowledge in sport-based settings (Hurley et al., 2018), researchers should explore the efficacy of training (e.g., what works, what does not work, why does it work, etc.).

A better understanding of factors contributing to mental health literacy will also help shape future interventions, whether for a coach or athlete. We must caution that future research should not focus solely on individual interventions to address mental health issues within sport. Instead, it must utilize the ecological framework to both understand issues and provide solutions at the system level in sports.

## Author Contributions

All authors contributed to this document. MM contributed 50%, JR contributed 20%, JD contributed 15%, KT contributed 10%, and GC contributed 5%. All authors contributed to the article and approved the submitted version.

## Conflict of Interest

The authors declare that the research was conducted in the absence of any commercial or financial relationships that could be construed as a potential conflict of interest.

## Publisher's Note

All claims expressed in this article are solely those of the authors and do not necessarily represent those of their affiliated organizations, or those of the publisher, the editors and the reviewers. Any product that may be evaluated in this article, or claim that may be made by its manufacturer, is not guaranteed or endorsed by the publisher.
